# Effect of Melatonin Implants during the Non-Breeding Season on the Onset of Ovarian Activity and the Plasma Prolactin in Dromedary Camel

**DOI:** 10.3389/fvets.2018.00044

**Published:** 2018-03-12

**Authors:** Khalid El Allali, Abdelmalek Sghiri, Hanan Bouâouda, Mohamed Rachid Achaâban, Mounir Ouzir, Béatrice Bothorel, Mohammed El Mzibri, Najia El Abbadi, Adnane Moutaouakkil, Ahmed Tibary, Paul Pévet

**Affiliations:** ^1^Comparative Anatomy Unit/URAC49, Department of Biological and Pharmaceutical Veterinary Sciences, Hassan II Agronomy and Veterinary Medicine Institute, Rabat, Morocco; ^2^Animal Reproduction Unit, Department of Medicine, Surgery and Reproduction, Hassan II Agronomy and Veterinary Institute, Rabat, Morocco; ^3^Harvard Medical School and Veterans Administration Healthcare System, Boston, MA, United States; ^4^Group of Research in Physiology and Physiopathology, Department of Biology, Faculty of Science, University Mohammed V, Rabat, Morocco; ^5^Neurobiology of Rhythms UPR 3212 CNRS, Institute for Cellular and Integrative Neurosciences, University of Strasbourg, Strasbourg, France; ^6^Biotechnology and Engineering of Biomolecules Unit, National Center of Energy, Science and Nuclear Techniques, Rabat, Morocco; ^7^Comparative Theriogenology, Department of Veterinary Clinical Science, College of Veterinary Medicine, Centre for Reproductive Biology, Washington State University, Pullman, WA, United States

**Keywords:** melatonin, ultrasonography, ovarian activity, dromedary camel, breeding season

## Abstract

To examine a possible control of reproductive seasonality by melatonin, continual-release subcutaneous melatonin implants were inserted 4.5 months before the natural breeding season (October–April) into female camels (Melatonin-treated group). The animals were exposed to an artificial long photoperiod (16L:8D) for 41 days prior to implant placement to facilitate receptivity to the short-day signal that is expected with melatonin implants. The treated and control groups (untreated females) were maintained separately under outdoor natural conditions. Ovarian follicular development was monitored in both groups by transrectal ultrasonography and by plasma estradiol-17β concentrations performed weekly for 8 weeks and then for 14 weeks following implant insertion. Plasma prolactin concentrations were determined at 45 and 15 days before and 0, 14, 28, 56, and 98 days after implant insertion. Plasma melatonin concentration was determined to validate response to the artificial long photoperiod and to verify the pattern of release from the implants. Results showed that the artificial long photoperiod induced a melatonin secretion peak of significantly (*P* < 0.05) shorter duration (about 2.5 h). Melatonin release from the implants resulted in higher circulating plasma melatonin levels during daytime and nighttime which persisted for more than 12 weeks following implants insertion. Treatment with melatonin implants advanced the onset of follicular growth activity by 3.5 months compared to untreated animals. Plasma estradiol-17β increased gradually from the second week after the beginning of treatment to reach significantly (*P* < 0.01) higher concentrations (39.2 ± 6.2 to 46.4 ± 4.5 pg/ml) between the third and the fifth week post insertion of melatonin implants. Treatment with melatonin implants also induced a moderate, but significant (*P* < 0.05) suppressive effect on plasma prolactin concentration on the 28th day. These results demonstrate that photoperiod appears to be involved in dromedary reproductive seasonality. Melatonin implants may be a useful tool to manipulate seasonality and to improve reproductive performance in this species. Administration of subcutaneous melatonin implants during the transition period to the breeding season following an artificial signal of long photoperiod have the potential to advance the breeding season in camels by about 2.5 months.

## Introduction

Photoperiod is an important environmental cue of seasonal reproduction control in several mammalian species. Day-length information changes are transmitted to the pineal gland to induce a nocturnal melatonin synthesis and a marked increase of its blood concentrations at night (1–3). Nocturnal melatonin secretion is proportional to night duration (4, 5). Mammals are informed about seasonal changes of photoperiod through the variations in the length of nocturnal peak of this hormone (6, 7). Therefore, a long duration of melatonin secretion provides a signal of a short-day/long night (short photoperiod: winter days); while a short duration of melatonin secretion informs on long day/short night (long photoperiod: summer days). Melatonin treatment has been used for manipulation of reproductive seasonality in several species. In ewes (8–13), goats (14–16), red deer (17), and mares (18), repeated melatonin injections or its constant release *via* implants are used during anestrus to induce early onset of reproductive cyclicity with a positive effect on ovarian activity and a suppressive effect of prolactin secretion.

The dromedary camel exhibits a seasonal reproduction (19, 20). In the Northern hemisphere, the mating season occurs in most cases during the short photoperiod matching low ambient temperatures, rainfall, and good food availability (20). In Morocco, the breeding season lasts from November to April (21). The relative importance of the environmental cues, as well as, the neuroendocrine mechanisms underlying seasonality in camel breeding is not fully elucidated. Seasonal variations of the photoperiod throughout the year affect the pattern of melatonin secretion in camels with a nocturnal peak during the short photoperiod (i.e., winter) (22). In a recent trial on lactating female camels, melatonin implants were shown to stimulate follicular development (23). These findings suggest that photoperiod is involved in regulation of reproductive seasonality and that melatonin may be used to manipulate the breeding season.

The female camel has a mating-induced ovulation and a gestation period lasting 12–14 months (20, 21). Therefore, mating and parturition occur in the same period of the year. Because of lactation anestrus, calving female camels have to wait until the following breeding season in order to conceive, which increases the calving interval and reduces reproductive efficiency (23). Elucidation of mechanisms regulating seasonality could lead to methods for advancement of the breeding season, decreased calving interval, and better distribution of calving throughout most of the year for provision of milk to the market. In addition, advancement of the breeding season would allow optimization of embryo production and genetic progress in an embryo transfer program.

The aim of this study was to determine the efficacy of melatonin implants as a method to advance the breeding season in the camel females. The main objective was to determine the effect of melatonin implant treatment for 4.5 months prior to the natural breeding season on onset of ovarian follicular activity. To achieve this goal, the following parameters were investigated: (1) the efficiency of melatonin release from the subcutaneous implants, (2) the pattern of ovarian follicular activity after melatonin treatment as assessed by ultrasonographic monitoring and circulating plasma estradiol concentrations, and (3) the suppressive effect of melatonin on prolactin plasma concentrations.

## Materials and Methods

### General

This study was carried out at the Comparative Anatomy Unit laboratory, Hassan II Agronomy and Veterinary Institute, Rabat, Morocco (latitude: 34°01′ N, 6°50′ W) during the non-breeding season (April–September). Twelve adult dromedary females (9–13 years of age) were randomly allocated to a control (*n* = 6) and melatonin-treated (MT group, *n* = 6) groups. Each female camel received a daily balanced diet consisting of 2 kg of a commercial feed (Alf Sahel^®^) and 3 kg of straw. Water was provided *ad libitum*.

Camels in the control group were maintained outdoors under natural environmental conditions for the entire experimental period. At the end of the natural breeding season and the beginning of the anestrous season (20th April), females in the MT group were exposed to an artificial long photoperiod (16L:8D) for 41 days. This treatment was performed to facilitate the receptivity of females to the following signal of short-day effect that is expected from using melatonin implants. After exposure to the long days artificial photoperiod, camels in the MT group were moved outdoors under natural conditions, but separated from the control group. On May 31st, each female in the MT group received 20 subcutaneous implants of melatonin of 18 mg each (melovin^®^, Ceva, Ltd., implant size: 3 mm). The melatonin dose used in this study (1 implant per 28 kg of body weight) was calculated based on a previous study on camels (23) and studies on mares (18). Each female received 20 implants (10 implants per ear) on the same day. Implants were placed using a standard implant syringe with a 12 gauge needle that is commercialized with the melatonin kit. Implant placement was tolerated by the animal without need for sedation or analgesia. The study was performed in conformity with the Hassan II Agronomy and Veterinary Institute of Rabat and Moroccan Ministry of Agriculture recommendations, which are in accordance with international ethical standards (24).

### Ultrasonographic Monitoring of Follicular Activity

Ovarian follicular activity was monitored in all females by transrectal ultrasonography using a 5 MHz frequency linear transducer (ALOKA SSD 550, Hitachi-Aloka Medical, Tokyo, Japan). The examinations were performed weekly for the first 8 weeks and for 14 weeks following melatonin implant insertion. Follicular activity was assessed by recording the diameter of all follicles present at each examination. Only mature follicles (diameter ≥1.0 cm) were considered for analysis (25, 26).

### Melatonin Assay

Plasma melatonin concentrations were determined to monitor the endogenous signal during different phases of the experiment. The aim of this assay was to compare changes in the peak of melatonin concentrations in the MT group under artificial long photoperiod (16L:8D) to those in the control group maintained under a natural photoperiod of spring (14L–10D). Blood samples were taken at intervals of 2 h during a 24 h period, on the 26th day (May 16th) after initiation of the artificial long photoperiod.

Melatonin was also assayed to assess the efficiency of delivery by subcutaneous melatonin implants. The contribution of the exogenous melatonin from the implants was evaluated by comparing daytime melatonin concentrations in the MT group with those of the control group. In addition, the 24 h rhythm of secretion of this hormone in the two groups was analyzed. Blood samples for daytime points comparison were collected at the same hour of the day (10:00 a.m.), 7 days before (−7), and weekly from day 0 (day of implant placement) until day 98 after melatonin implant insertion. The 24-h melatonin rhythm in the two groups was assessed on blood samples collected at intervals of 2 h on the 28th day after melatonin implant insertion.

Blood samples were collected in heparinized vacutainer tubes, alternately from the right and left jugular veins, and immediately centrifuged at 1,760 *g* for 30 min. Plasma was then harvested and stored at −20°C for subsequent melatonin assay.

Camel plasma melatonin concentrations were determined using a direct solid-phase radioimmunoassay as described previously (22). Melatonin was extracted by using dichloromethane. Duplicate aliquots containing each 100 µl of extracted blood plasma were assayed by adding 100 µl of a specific rabbit antiserum (R19540, INRA, Nouzilly, France) and 300 µl of labeled [^125^I]-2-iodomelatonin. The mixture solution was incubated overnight. Then 800 µl of an anti-rabbit c-globulin were added and tubes were kept refrigerated within ice for 1 h. Tubes were then centrifuged and the supernatant discarded. The pellet was counted using γ-ray counter (COBRA CPM Model 5002, Packard Instrument Company, USA).

### Estradiol-17β and Prolactin Assays

Blood samples for the estradiol-17β assay were taken at the same time (10:00 a.m.) on the days of ultrasonographic ovarian examination. Plasma prolactin concentrations were determined in both groups of camels on blood samples taken 45 and 15 days before and then on days 0, 14, 28, 56, and 98 after melatonin treatment (Day 0).

Plasma estradiol-17β and prolactin hormones were determined using quantitative Sandwich ELISA kits (MyBioSource Inc., San Diego, CA, USA), respectively, Camel estradiol-E2 ELISA Kit (Catalog No. MBS090600) and Camel Prolactin Luteotropic Hormone (PRL/LTH) ELISA Kit (Cat.No: MBS089793). The MBS090600 is a ready-to-use microwell, strip plate kit for analyzing the presence of estradiol-17β in the camel biological samples, including the plasma. The kit is based on the interactions between the estradiol-17β antibody and the estradiol-17β antigen (immunosorbency) and a horseradish peroxidase colorimetric detection system to detect estradiol-17β antigen targets in the tested samples. The concentration gradients of the kit standards give a corresponding detection range of 15.6–500 pg/ml, with a sensitivity of the assay of 2.0 pg/ml. The quality control assays of the kit provide intra- and inter-assay coefficients of variations each less than 15%. The intra- and inter-assay coefficients of variation calculated for the assay of estradiol-17β in this study were 10.4 and 8.2%, respectively. The MBS089793 is also a ready-to-use microwell strip plate Sandwich ELISA Kit for analyzing the presence of the Prolactin in camel biological samples. The technique used is the same as described above for the MBS090600 kit. The kit detection range is 0.625–20 ng/ml with an estimated sensitivity of 0.1 ng/ml. The kit quality control assays evaluating reproducibility provide intra- and inter-assay coefficients of variations each less than 15%. The intra- and inter-assay coefficients of variation calculated for the assay of prolactin in this study was 8.4 and 6.5%, respectively.

### Statistical Analysis

Ovarian follicular activity was expressed as the percentage of females presenting mature follicles (diameter >1.0 cm). Values of plasma melatonin, estradiol-17β, and prolactin concentration were expressed as means ± SEM.

Melatonin data for the artificial accelerated long photoperiod (16L:8D) were analyzed using nonlinear regression. The curve representing the secretion profile was fitted to the curve generated by the following equation (27):

$$f=y_{0}+[y_{\text{ampl}}/\{\text{l}+\text{exp}[\text{slope1}*(\text{phi1}-x)]\}*\{\text{1}+\text{exp}[\text{slope2}*(x-\text{phi2})]\}]$$

where *f* is the concentration at the *n*th data point, x is the time of this *n*th data point, *y*_0_ is the basal level measured during daytime, *y*_ampl_ is the amplitude of the nocturnal peak, phi1 and phi2 are the inflection points and represent the time points at which 50% of the maximal increase and 50% of the decline were reached, respectively. Slope1 and slope2 are the slopes of the onset and of the decline of the peak.

The duration of the peak secretion was calculated as the difference between phi1 and phi2. The differences in melatonin amplitude and duration of the peak secretion between treated and controls camels were compared using Student’s *t*-test, *via* Statistical Software (SPSS) version 14.0 for Windows (SPSS, Inc., Chicago, IL, USA).

Estradiol-17β and prolactin data were analyzed using the same software. Simple one-way analysis of variance was used to compare the means of plasma concentrations of these hormones in both MT and control groups. The differences in melatonin concentrations were compared point by point.

## Results

### Plasma Melatonin Concentrations

**Figure 1:**
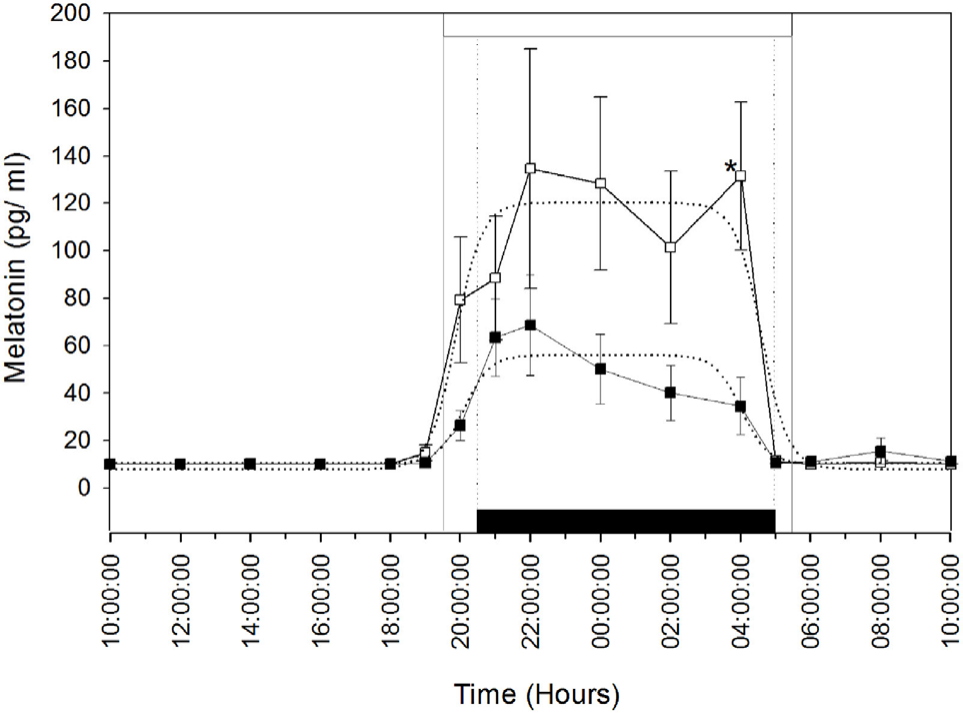
Rhythm of melatonin secretion in camels [Melatonin-treated (■) and control groups (□)] during 24 h on May 16th: day 26 after the establishment of the artificial long photoperiod (16L: 8D) in the melatonin-treated group; the control group remained under outdoor natural photoperiod. The black bar corresponds to the duration of darkness of LD-cycle during the artificial long photoperiod of the melatonin-implanted group and the white bar is the duration of night in the outdoor natural conditions of the control group. Values are expressed as mean ± SEM. The significance levels for differences between treatments (ANOVA) are indicated: *P < 0.05.

Plasma melatonin concentrations were determined in the MT group after the artificial long photoperiod to make sure that they integrated this signal. The mean (±SEM) and profiles of plasma melatonin are shown in Figure 1. Circulating melatonin concentrations were below detectable levels (<10 pg/ml), during the daytime then increased gradually after the beginning of the scotophase in both groups of females. Plasma melatonin concentrations showed individual variations. The nocturnal plasma concentrations, from 20:00 p.m. to 4:00 a.m., remained lower in the MT group compared to the control group; however, the difference was statistically significant only at 4:00 a.m. (*P* < 0.05). Plasma melatonin concentrations declined thereafter (from 4:00 a.m.) in both groups.

The means (±SEM) of the individual phi1 and phi2 estimated by the nonlinear regressions are reported in Table 1. There was no significant difference in the onset (phi1) and the decline (phi2) of melatonin release. Moreover, the amplitude (*y*_ampl_) of the melatonin peak was not significantly different between the two groups. However, the duration of the melatonin peak was significantly (*P* < 0.05) shorter, by 2.5 h, in the MT group compared to that of the control group. These results confirm that the artificial long photoperiod was effective in providing long-day signal prior to melatonin implant insertion.

**Table 1:** Mean values ± SEM of individual phi1 and phi2, amplitude, and duration of the melatonin secretion peak in melatonin-implanted and control groups.

	Control group (*n* = 6)	Melatonin group (*n* = 6)	Statistics
phi1phi2	20.68 ± 0.4126.51 ± 0.94	20.09 ± 0.3928.48 ± 0.12	*P* = 0.336*P* = 0.066
Amplitude of melatonin secretion peak (*y*_ampl_)	55.40 ± 14.56	126.01 ± 34.94	*P* = 0.092
Duration of melatonin secretion peak	5.83 ± 0.45	8.38 ± 1.10	*P* = 0.029*

*P < 0.05.

**Figure 2:**
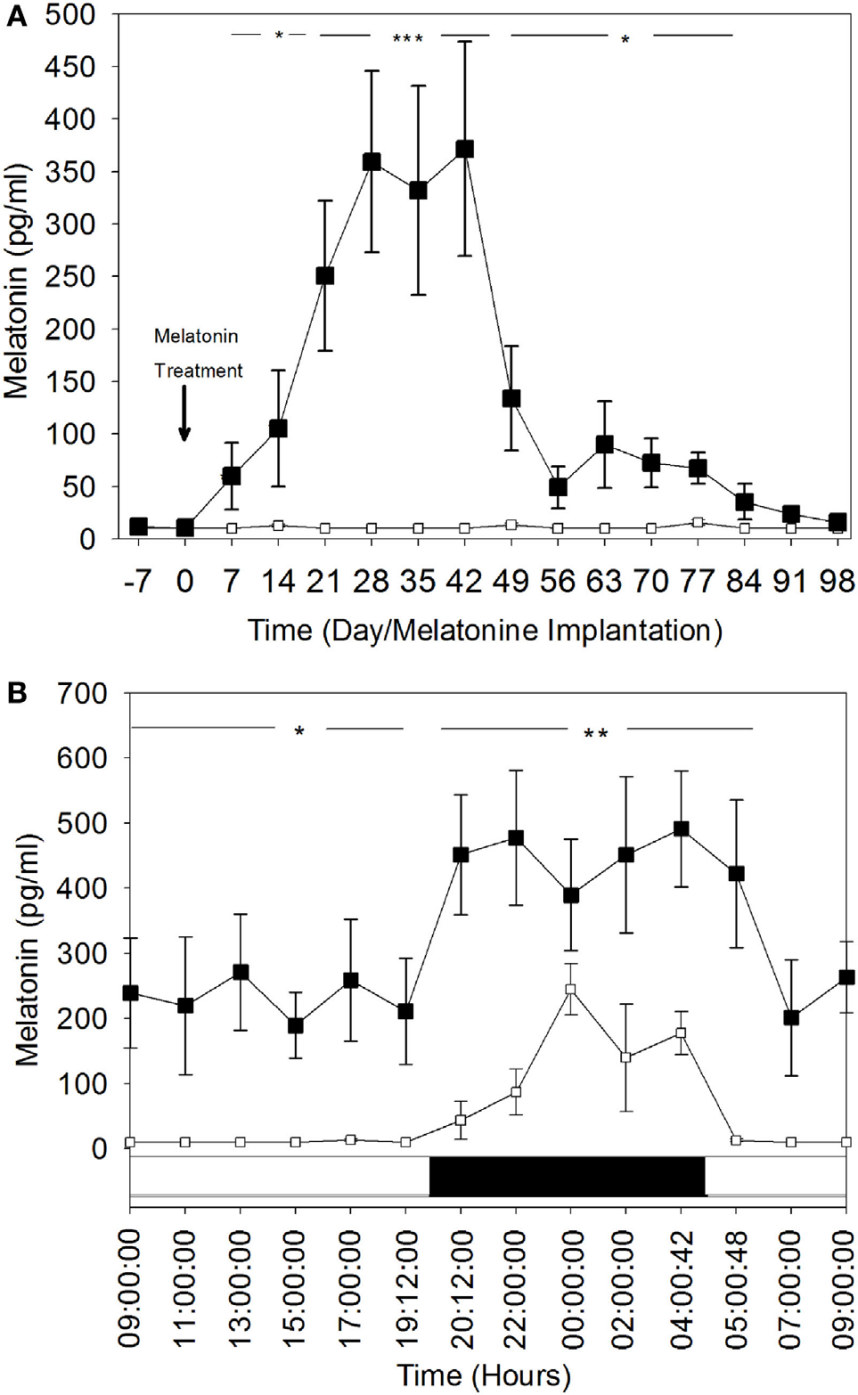
Comparison of plasma melatonin concentrations in melatonin-treated group (■) and the control group (□). **(A)** Day time plasma melatonin concentrations (mean ± SEM) before and after insertion of melatonin implants. Blood was sampled every week during the day and at the same hour: 10:00. **(B)** Comparison of 24 h rhythm of plasma melatonin concentrations (mean ± SEM). The blood was sampled during the 28th day after melatonin implants insertion. Bottom white and black bars: durations of the day and the night. The significance levels for differences between treatments (ANOVA) are indicated: *P < 0.05, **P < 0.01, and ***P < 0.001.

Weekly plasma melatonin concentration variations during the day-time are shown in Figure 2A. During the entire experimental period (i.e., 15 weeks), the mean (±SEM) daytime plasma melatonin concentrations in the control group remained below or at the limit of detectable levels (10 pg/ml). In contrast, these concentrations were significantly elevated in the MT group and increased significantly (*P* < 0.05) by the end of the first week after melatonin implant insertion to reach very high levels (331.9 ± 99.7 and 371.7 ± 102.3 pg/ml) between the fourth and the sixth weeks after treatment (*P* < 0.001). After the sixth week, daytime plasma melatonin concentrations declined gradually, but remained significantly higher than those in the control group.

These findings were confirmed by the hourly plasma melatonin concentrations for the 24-h rhythm on the fourth week (Day 28 after melatonin treatment) (Figure 2B). Both diurnal and nocturnal plasma melatonin concentrations were significantly high (*P* < 0.05). Daytime plasma melatonin concentrations in the MT group were significantly higher than the nocturnal plasma melatonin levels of the control group. The nocturnal levels of melatonin in MT females were increased significantly, which shows that the light–dark cycle was still affecting plasma melatonin concentration in this group.

### Ovarian Activity and Plasmatic Estradiol-17β Concentrations

**Figure 3:**
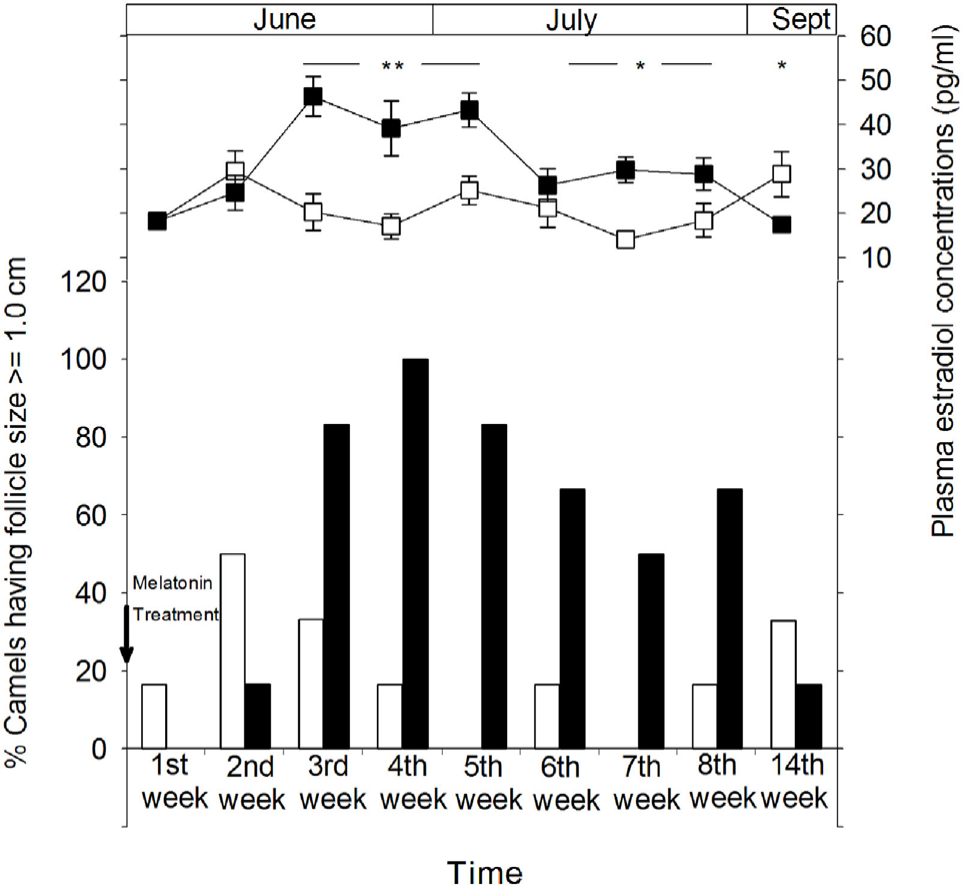
Ovarian cyclical activity in camel females of the melatonin-treated group (■) and control group (□) determined by sonographic monitoring of the follicular diameter and plasma estradiol concentrations. The figure shows the percentage of camels with a follicle size of ≥1.0 cm. Time is presented as days relative to insertion of melatonin implants. The significance levels for differences between treatments (ANOVA) are indicated: *P < 0.05, **P < 0.01, and ***P < 0.001.

Figure 3 shows the percentage of female camels of both MT and control groups displaying ovulatory follicles (≥1.0 cm). The percentage of females presenting ovulatory follicles is higher in the MT group than that of the control group. However, the presence of mature follicles in a small proportion of the females in the control group indicates that ovarian activity is present in some animals after the breeding season. The highest percentages (83.3%) of females exhibiting follicles of more than 1.0 cm were observed 1 month after melatonin treatment. 14 weeks later (in September), these percentages decreased in the MT group to reach approximately those of the control group, 16.6 (1/6) and 33.3% (2/6), respectively. It is important to note that no large anovulatory follicles were identified throughout the experimental period.

Ovarian follicular activity observed by ultrasonography was positively correlated with plasma concentrations of estradiol-17β in both groups (Figure 3). In the MT group, plasma estradiol concentrations increased gradually from the second week after melatonin treatment to reach significantly (*P* < 0.01) higher levels ranging between 39.2 ± 6.2 and 46.4 ± 4.5 pg/ml between the third and the fifth week of treatment and remained significantly (*P* < 0.05) higher until at least 2 months after treatment. However, on the 14th week (September), plasma estradiol-17β concentrations were significantly (*P* < 0.05) lower than that of the control group (17.5 ± 1.3 vs 28.9 ± 5.1 pg/ml).

### Melatonin Effect on the Plasmatic Pattern of Prolactin

**Figure 4:**
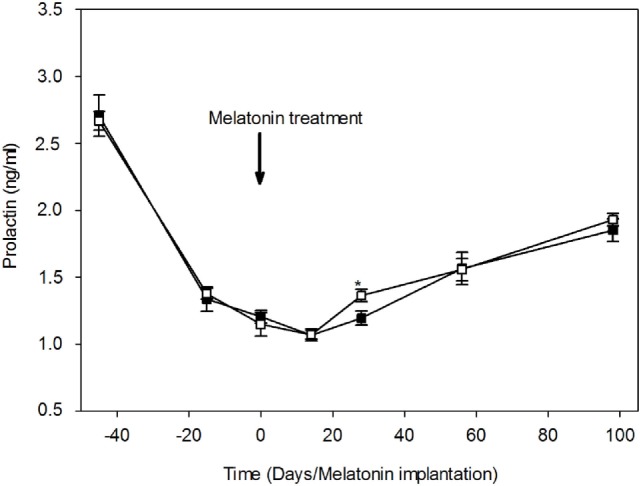
Variations of plasma prolactin concentrations (mean ± SEM) in camel females of the melatonin-treated group (■) and control group (□). Time is expressed as days relative to the melatonin implants insertion: before and after day 0. At day 28, prolactin concentration was significantly (*P < 0.05) lower in the melatonin-treated group compared to the control group.

Plasma prolactin concentrations throughout the study are shown in Figure 4. Plasma prolactin concentrations were relatively higher in both groups (2.71 ± 0.15 and 2.67 ± 0.07 ng/ml in MT and control group, respectively) during the transition period between the breeding and non-breeding season (April 20th). 1 month later, plasma concentrations of prolactin declined dramatically in both groups, indicating a seasonal effect. Plasma prolactin concentrations reached a minimum (1.07 ± 0.03 and 1.07 ± 0.04 ng/ml, in the MT and control group, respectively) around the summer solstice (June14th). After the summer solstice, plasma prolactin concentrations increased progressively to reach a mean value of 1.85 ± 0.08 ng/ml in the MT group and 1.93 ± 0.05 ng/ml in the control group on September (98th day after treatment). The point to point comparison throughout the study shows that plasma prolactin concentrations were significantly (*P* < 0.05) lower in the MT group (1.19 ± 0.05 ng/ml) compared to the control group (1.36 ± 0.05 ng/ml) at 28th day after treatment. The profile of plasma prolactin was similar in both groups.

## Discussion

Results of this study illustrate clearly that the constant release of exogenous melatonin from implants was able to affect ovarian activity and prolactin concentration patterns in female camels. These effects are consistent with the major function of melatonin that is conveying the signal of photoperiod change to the brain and the body. Melatonin is a hormone that informs on the timing of day and season and regulates the waxing and waning of seasonal reproductive function in mammals [for review see Ref. (28)]. It is well known that it is the duration of the daily melatonin secretion that encodes the day-length information rather than its amplitude (6, 7). Therefore, the exogenous melatonin provides a long duration of circulating plasma melatonin, inducing a short-day signal. Our results show that melatonin implants provide a continuous release of melatonin as demonstrated by the long duration and high levels of circulating plasma melatonin during day time for 12–14 weeks after treatment. Melatonin release by the implant decreased significantly after the 14th week. This duration of melatonin release from the implants is within the expected range of 70–90 days indicated by the manufacturer based on previous studies (29, 30). This may explain the decrease in ovarian activity in melatonin-treated females in September.

In camels, annual photoperiodic changes were shown to be integrated and secretion of melatonin increased steeply after sunset and remained higher throughout the night (22, 31). In addition to the high daytime concentrations of melatonin observed in this study, the nocturnal plasma melatonin concentrations were also significantly elevated in the melatonin-treated females. These results suggest that the endogenous melatonin from the pineal gland released during the night is involved in the overall increase of circulating melatonin levels in addition to release from the implant. This finding indicates that the light-dark cycle continues to exert its effect on the brain, despite continuous melatonin release from the implants. Similar daytime levels of circulating melatonin were observed with subcutaneous implants in other species [sheep (11); deer (17); horses (32)]. However, the effects of exogenous melatonin treatment differ depending on species, melatonin implant concentration, and time of treatment (11). In the camel, there is a paucity of information on the role of melatonin on reproduction. Only one previous study attempted induction of follicular activity in lactating camels with melatonin treatment (23). However, the treatment in that study was initiated 2 months prior to the breeding season which is too close to the natural breeding season. In this study, we decided to start the melatonin treatment earlier in order to determine its effect on ovarian activity. Plasma melatonin concentrations revealed that the duration of the secretion peak was shorter under the artificially long photoperiod, which is in accordance with the results of our previous studies concerning the neuroendocrine ability of the camel to integrate the photoperiodic changes (22, 31). The melatonin treatment protocol used in this study led to a stimulation of the ovarian cyclicity during the non-breeding season and 3.5 months earlier onset of ovarian activity. This is illustrated by the significant increase in the percentage of females exhibiting a mature follicle and a significant rise of plasma estradiol-17β concentration in the melatonin-treated females compared to the control group.

The camel is an induced ovulator species. In absence of mating, overlapping follicular waves of variable duration occur (25, 26, 33). Occasionally, large anovulatory follicles may develop in some females, but they do not seem to affect follicular activity. In this work, no anovulatory follicles were observed. The presence of mature follicle with size ≥1.0 cm was evident in the MT group for at least 5 weeks, starting from the third week after treatment. This can be related to the partial overlap of two successive follicular waves.

The ovarian follicular stimulation observed in the MT group was associated with a significant increase in plasma estradiol-17β concentrations. It is well established that estradiol-17β is the main hormone that characterizes the ovarian status in the camel and is positively correlated with follicular size (25, 33–35). Basal blood concentrations of estradiol are around 25 pg/ml (36, 37). As the follicles grow, estradiol concentration increases to reach high levels of 39 pg/ml when the dominant follicle measures 1.7 cm (25, 37) or higher (36), then declines when the follicle exceeds the diameter of 1.7 cm (25) or is in the regression phase (38). Seasonal variations were also reported in the camel, with significant estradiol levels during the breeding season (34, 39). The recorded plasma estradiol-17β concentrations in this study are within the reported range for normal follicular activity. The estradiol-17β and ovarian follicular activity found in our study suggest that the MT group displayed all characteristics of a seasonal breeding during the anestrus period of the summer.

Melatonin implant treatment of female camels in the early anestrus season induced significant effects on the ovarian activity and plasma estradiol-17β concentrations and on the prolactin secretion from the adenohypophysis. Plasma prolactin concentrations measured in this study did not exceed 2.8 ng/ml and seem to be very low compared to other ruminants, such as goats (15), mouflon, and sheep (40). These results are consistent with those of previous studies that show low concentrations of plasma prolactin in camels (41, 42).

Melatonin is known for its effect on prolactin secretion which seems to be mediated by the pars tuberalis (8, 11, 43). This pars tuberalis contains the largest component of melatonin receptors MT1 (44–46), giving evidence of its involvement in the melatonin control of the seasonal prolactin secretion. Prolactin is also known to exhibit a seasonal rhythm in several photoperiodic seasonal breeders (47), showing a negative relationship to the seasonal rhythm of melatonin (48, 49). In this study, both MT and control groups had high levels of plasma prolactin during the transition period between the breeding and the non-breeding seasons. Plasma prolactin concentrations declined dramatically in both groups until the onset of the summer solstice and then increased progressively until the end of the experiment in September. These variations suggest the presence of a seasonal rhythm of prolactin in the camel, which was proposed previously by other authors (42, 50, 51). Our results on camel prolactin patterns are close to those observed in goats, with a similar decrease of plasma concentrations in early summer followed by an increase in early Fall (15, 52).

Prolactin concentrations were significantly lower in the MT group 28 days after initiation of treatment. This result was expected due to the well-known suppressive effect of melatonin as mentioned above. The high plasmatic concentrations of melatonin from the implant have a simultaneous effect on prolactin, gonadotrophins, and ovarian activity. Melatonin implants elicit a significant increase of LH/FSH secretion and thus a gonadic stimulation, but also a suppressive effect on the prolactin secretion [For review see Ref. (53)]. The high concentrations of melatonin released from the implants seem to act directly on pars tuberalis (8, 11, 43) to suppress the prolactin, but also seem to act on this pars (53) to initiate a reaction in the medio-basal hypothalamus leading to the activation of the gonadotrophic axis.

Mechanisms in the medio-basal hypothalamus involved in the melatonin control of reproduction were actively investigated during past decades. The fixation of melatonin on MT1 receptors of the pars tuberalis induces high levels of thyroid-stimulating hormone synthesis during long photoperiod (53). Subsequently, the tanycytes activate TSH receptors and induce the production of deiodinase 2 which activate within the medio-basal hypothalamus, the conversion of thyroxine to triiodothyronine (T3) (53–55). Moreover, the thyroid-stimulating hormone increases the expression of two neuropeptides recently described to be involved in the control of seasonal breeding, the hypothalamic RFamide-related peptide (RFRP), and Kisspeptin peptides (56, 57). Kisspeptin and RFRP, which are regulated by melatonin concentrations depending on the species and photoperiod control, act directly on the GnRH neurons to regulate the synthesis of LH and FSH responsible for the onset and offset of reproduction season (58–62). From these results, it can be concluded that the control of seasonal breeding by the melatonin-thyroid-stimulating hormone T3 occurs *via* these neurons (55, 63).

The decline in plasma prolactin concentration in the MT group appears to be moderate when compared to results obtained in ewes (11, 64), goats (15), and mouflon (43). It is well established that this hormone stimulates mammary gland development and regulation of lactation. In human and rodents, prolactin also intervenes in the control of ovarian steroidogenesis, formation of corpus luteum, and modulation of the effects of gonadotropins (65). The observed low concentrations of prolactin over 2 months coincide with high ovarian activity and estradiol synthesis in the MT group and with a low gonadal activity in the control group. This indicates that prolactin did not exert a decisive role in the regulation of the breeding season. A similar observation was reported in the goat (15), but not in the ewe (64, 66, 67), in which prolactin drives the ovarian activity. However, prolactin is particularly known for its effect on other seasonal rhythms, such as hair growth and molting (47, 68) rather than a direct effect on the breeding season and reproduction.

The results of the present experiment provide evidence that seasonality of reproduction in the dromedary camel is at least partially controlled by photoperiod through the same mechanisms described in other seasonal breeders, such as small ruminants and horses. It is also clear from the results obtained in the control group that a significant proportion of dromedary females continue to have ovarian follicular activity throughout the year. This is also not uncommon in other seasonal breeders. Individual genetic variation and breed effect may be involved in the response to seasonal variation in the photoperiod. It is important to note that male camels appear to be more seasonal than female camels. This is a unique phenomenon in domestic animal species. Other external factors, such as nutrition, temperature, and presence of males may play an important role in female seasonality. Further research on the effect of these factors and their interrelationship is needed in order to elucidate the mechanism regulating seasonality in the dromedary.

## Ethics Statement

The study was performed in conformity with the Hassan II Agronomy and Veterinary Institute of Rabat and Moroccan Ministry of Agriculture recommendations, which are in accordance with international ethical standards (24).

## Author Contributions

KA, PP, and AT conceived and designed the work. KA and HB conducted the experiment and performed sampling. AS performed ultrasonography monitoring. KA, MO, BB, MM, AM, and NA performed hormonal assays and analysis. KA prepared the manuscript. AS, HB, MA, MO, BB, MM, AM, NA, AT, and PP revised and approved the final review.

## Conflict of Interest Statement

The authors declare that the research was conducted in the absence of any commercial or financial relationships that could be construed as a potential conflict of interest.
